# *Capnocytophaga sputigena* Causing Complicated Sinusitis With Intracranial Involvement in a Pediatric Patient

**DOI:** 10.1155/crot/7154542

**Published:** 2025-06-19

**Authors:** Karan Gandhi, Chloe Pulver, Lucas D. D. Foster, David Plemel, Nancy Nashid, Keng Tay, Julie E. Strychowsky

**Affiliations:** ^1^Department of Otolaryngology-Head and Neck Surgery, Western University, London, Ontario, Canada; ^2^Department of Ophthalmology, Western University, London, Ontario, Canada; ^3^Department of Pediatrics, Western University, London, Ontario, Canada; ^4^Department of Radiology, Western University, London, Ontario, Canada

**Keywords:** acute bacterial rhinosinusitis, *Capnocytophaga sputigena*, intracranial complications, multidisciplinary care, pediatric

## Abstract

This report presents a rare case of acute bacterial rhinosinusitis with orbital and intracranial complications caused by *Capnocytophaga sputigena* in a pediatric patient. A 15-year-old male presented with orbital cellulitis, acute sinusitis, and meningismus. Brain imaging showed evidence of early intracranial abscess formation and areas of cerebral infarction. He underwent urgent endoscopic sinus surgery (ESS) and drainage of an orbital subperiosteal abscess. This case underscores the critical role of timely diagnostic imaging, multidisciplinary care, appropriate surgical management, and effective culture-directed antimicrobial therapy in treating sinusitis and its complications.

## 1. Introduction

Acute rhinosinusitis (ARS) lasts for less than 4 weeks, is primarily viral in origin, and typically resolves spontaneously [1]. Acute bacterial rhinosinusitis (ABRS) occurs in a small subset of patients and should be suspected when symptoms persist for more than 10 days or worsen after initial improvement [2]. ABRS is predominantly caused by *Streptococcus pneumoniae*, *Haemophilus influenzae*, and *Moraxella catarrhalis* in the pediatric population [2, 3]. ABRS can also lead to orbital and intracranial complications. With the increasing prevalence of drug-resistant organisms, obtaining a specimen for microbiologic cultures is of utmost importance. Complicated ABRS necessitates a multidisciplinary approach involving otolaryngology, infectious disease specialists, ophthalmology, neurosurgery, neurology, and general pediatrics for effective management.

*Capnocytophaga species* are a group of slow-growing, facultative anaerobic gram-negative bacteria. Members of this genus can cause serious life-threatening infections including septicemia and meningitis*. Capnocytophaga sputigena* is part of the normal human oropharyngeal flora. While *C. sputigena* rarely causes infection in the general population, it has been implicated as an opportunistic pathogen, particularly in immunocompromised hosts [4]. To our knowledge, there is only one other reported case of *C. sputigena* causing sinusitis and a subperiosteal abscess [5]. In this case report, we present the first known case of *C. sputigena* contributing to orbital and intracranial complications secondary to ABRS.

## 2. Case Report

A 15-year-old, previously healthy, fully immunized male presented with a one-week history of right periorbital swelling and erythema accompanied by worsening headache, neck stiffness, and photophobia. This was preceded by a viral upper respiratory infection 2-3 weeks prior. He was afebrile at presentation but spiked fever of 38.6 degrees Celsius following admission. He was confused and unable to answer standard questions. His neurological exam showed left pronator drift and left-sided neglect. He had increased pain with neck flexion and positive Brudzinski's sign. Visual acuity was 20/50 in the right eye and 20/40 in the left eye. Pupils were equal and reactive to light without relative afferent pupillary defect (RAPD). Extraocular movements showed restricted adduction in the right eye. Flexible nasopharyngoscopy revealed purulence in the right middle meatus. Laboratory tests showed elevated white blood cell count (WBC) of 24.5 with lymphopenia (0.9) and C-reactive protein (CRP) level of 136.5. Empiric IV antimicrobial therapy was started with ceftriaxone, vancomycin, and metronidazole at meningitic doses. The patient was admitted and started on normal saline nasal rinses, intranasal corticosteroid spray, and a nasal decongestant.

**Figure 1:**
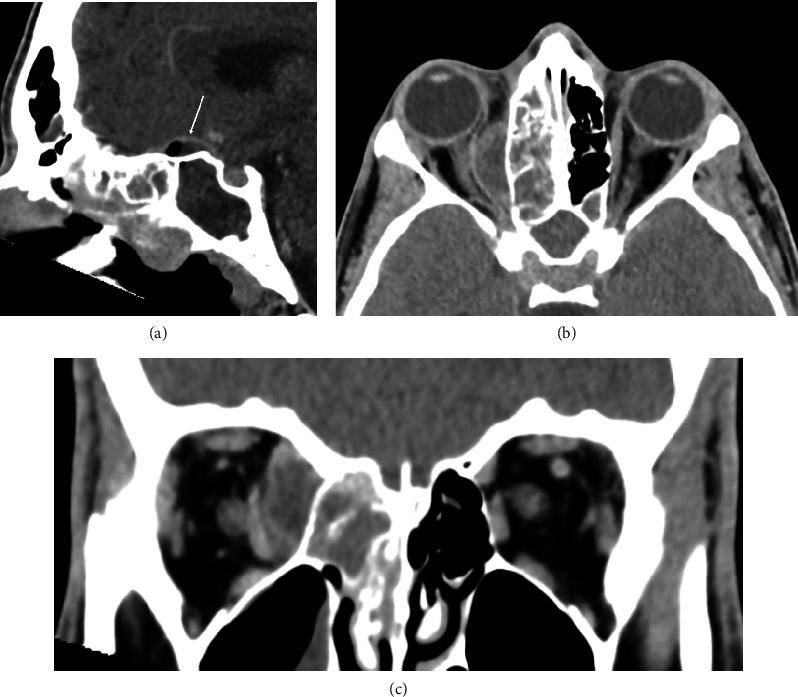
Sagittal view of extra-axial rim enhancing fluid with air locule indicates communication with the paranasal sinuses (a), axial view of the subperiosteal abscess (b), coronal view of subperiosteal abscess (c).

Computed tomography (CT) with contrast showed sphenoethmoidal sinusitis, a subperiosteal abscess along the right medial orbital wall, and a fluid collection superior to the planum sphenoidale suggestive of an early intracranial abscess (Figure 1). Focal lucencies in the right posterior limb internal capsule/thalamus indicated a potential infarct from affected arteries.

The patient underwent urgent endoscopic sinus surgery (ESS) and endonasal drainage of the subperiosteal abscess. The intraoperative fluid and tissue cultures showed *Capnocytophaga sputigena and Streptococcus anginosus*. A subsequent lumbar puncture showed low glucose 1.3 and elevated protein 1194, and pleocytosis WBC 96, RBC 3. CSF cultures did not have any growth after 5 days. Respiratory viral PCR was negative. SARS-CoV-2 antibodies were positive. Of note, the patient had blood cultures drawn at the referring hospital, but not in media intended for isolating anaerobic bacteria. Blood cultures were slow to grow; one had *Streptococcus intermedius* and the other showed positive gram stain but failed to grow in culture.

**Figure 2:**
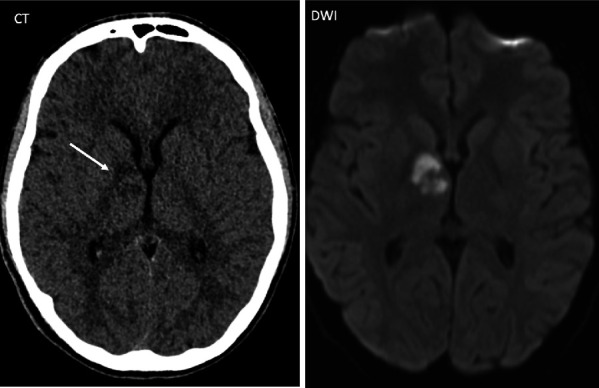
MR head done postoperatively showing infarctions in the right globus pallidus and anterior thalamus.

**Figure 3:**
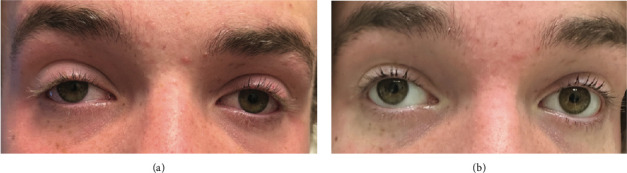
Demonstrates the patient's clinical appearance before (a) and after (b) apraclonidine testing. The right pupil enlarged from 2 mm to 4 mm, and the margin reflex distance one increased from 4 mm to 6 mm.

Postoperative MRI on day zero showed signal abnormalities consistent with infarcts including FLAIR hyperintensities in the right globus pallidus, anterior thalamus, medial temporal lobe, optic tract, and optic chiasm (Figure 2). CT on postoperative day 4 showed a nonocclusive filling defect in the cavernous sinus, suggestive of cavernous venous sinus thrombosis (CVST). The patient was discharged on postoperative day 15. Discharge medications included acetylsalicylic acid, normal saline rinses, intranasal corticosteroid spray. He completed a total of 6 weeks of antibiotics with intravenous (IV) ceftriaxone and oral metronidazole. Subsequent ophthalmological examination revealed a right Horner's syndrome and a left homonymous hemianopsia (Figure 3).

At his most recent follow-up, the patient was conscious, alert, and able to respond appropriately. He presented with continued fatigue, photosensitivity, decreased vision, and short-term memory loss. Visual field testing was not attempted due to photophobia and patient preference.

## 3. Discussion

ABRS complications are more common in children and those who are immunocompromised. About one-quarter of children with ABRS will have orbital complications including cellulitis or abscess formation [6]. Presenting signs and symptoms can include periorbital edema and erythema, proptosis, gaze impairment, limited extraocular motility, diplopia, and pupillary defects [7]. A smaller percentage of children with ABRS will have neurological deficits with intracranial complications [6, 8]. The frontal sinus is most implicated in these cases, but multiple sinuses can be involved [9]. Intracranial complications of pediatric sinusitis include empyema (49%), epidural abscess (36%), cerebral abscess (21%), meningitis (10%), cavernous sinus thrombosis (5.6%), osteomyelitis (3.3%), encephalitis (1.7%), and cerebral infarcts (1.1%) [9]. The patient in this case had a filling defect on CT imaging and a right Horner's syndrome, both suggestive of a CVST. The CVST likely caused septic emboli, leading to the multi-territorial strokes seen on postoperative MRI and the resultant left homonymous hemianopsia. Early signs and symptoms of intracranial involvement include headache, fever, nausea and vomiting, neurologic deficits, mental status change, seizures, and tender swollen forehead [9]. Urgent multidisciplinary care is needed for the management of ABRS complications.

A common management dilemma in complicated ABRS is the need for surgical intervention. The Chandler Classification is used to describe the extent of orbital complications in acute sinusitis. However, the Chandler classification does not indicate who requires sinus surgery. Chang et al. showed that some patients with Chandler Stage I undergo surgery and, while an increasing number of higher-Stage patients need surgery, some Stage V patients did not undergo surgery [10]. Several studies have explored factors predicting the need for surgical intervention in orbital infections secondary to sinusitis. Oxford et al. found that normal visual acuity, pupil function, an absence of ophthalmoplegia, intraocular pressure under 20 mmHg, proptosis less than 5 mm, and abscess width less than 4 mm on CT scans suggest a subperiosteal abscess may be managed medically [11]. Yosefof et al. found that the presence of a fever, ophthalmoplegia, diplopia, and higher Chandler classifications significantly increased the likelihood of surgery [12]. Main surgical indications included no response to IV antimicrobial treatment or worsening symptoms [12]. Saltagi et al. found that patients with subperiosteal abscesses from sinusitis were more likely to need surgery if there were serious ophthalmologic symptoms such as reduced visual acuity, RAPD, or restricted eye movements [13]. In patients with orbital cellulitis, Aryasit et al. found that abscess volume and initial visual acuity as key indicators for surgery [14]. They noted a 1514 mm^3^ volume threshold for abscess drainage with 71% sensitivity and 80% specificity and found that initial visual acuity below 20/200 significantly predicted poorer post-treatment vision, alongside RAPD as a predictor for worse visual outcomes [14]. When in doubt, surgery should be undertaken, as these cases pose a risk to vision. Patt et al. found 10% of their patients with subperiosteal abscesses secondary to sinusitis, and one of two with an orbital abscess experienced permanent vision loss [15]. Collaboration with ophthalmology and radiology can help guide the decision to proceed to surgery in borderline cases.

The approach for subperiosteal abscess drainage depends on the location of the abscess. Medial wall subperiosteal abscesses can be appropriately addressed via an endonasal approach during ESS for sinusitis management [16]. Migirov et al. found no complications in patients undergoing endonasal subperiosteal abscess drainage while those undergoing open surgery were more likely to have scarring and delayed healing [17]. Superior orbital subperiosteal abscesses are more nuanced; ones located medially may be accessible via an endonasal approach while those located more laterally often required an open surgery [18]. Combination surgery, including both an otolaryngologist and an oculoplastic surgeon, may be the safest method by which to address superior subperiosteal abscesses.

*Capnocytophaga sputigena* is rarely isolated in cases of bacterial sinusitis. A potential explanation for the mechanism of infection could be through direct extension from the oral cavity to the nasal passages. Since this pathogen is more often implicated in infections in immunocompromised patients, it would be imperative to rule out an underlying primary immunodeficiency. In our case, an immune work up was pursued but did not yield any findings. However, the preceding history of an upper respiratory tract infection and positive SARS-CoV-2 antibodies indicated a possible recent COVID-19 infection. It has been established that SARS-CoV-2 can cause lymphopenia and immune dysregulation, which might place a host at increased risk of infections and hinder their immune response to pathogens. This offers a potential explanation for the severity of this patient's infection.

The antimicrobial regimen for infections caused by *C*. *sputigena* should include either a third-generation cephalosporin, carbapenem, or a combination of beta-lactam and beta-lactamase inhibitor. Resistance to fluoroquinolones such as ciprofloxacin and levofloxacin, as well as to trimethoprim-sulfamethoxazole, is frequently observed. Clindamycin remains a viable alternative, in patients who cannot tolerate other options [19].

## 4. Conclusion

This is the first known pediatric case of *C. sputigena* contributing to a presentation of complicated bacterial sinusitis with orbital and intracranial complications, and only the second to implicate it in orbital complications. It underscores the importance of multidisciplinary management in treating ABRS with orbital and intracranial complications. Prompt diagnostic imaging, broad-spectrum antimicrobial therapy, and timely surgical intervention with cultures sent to establish a microbiologic diagnosis are paramount in the treatment of patients with complicated polymicrobial bacterial sinusitis where this organism could be a contributing culprit. An immune work up to rule out an underlying immunodeficiency should be considered. Finally, our understanding of the long-term effects of COVID-19 on immune function is still in evolution. Atypical pathogens need to be considered in infections with severe presentations or poor clinical response.

## Data Availability

Data sharing is not applicable to this article as no new data were created or analyzed in this study.
